# Cardiovascular outcomes in patients with co-existing coronary artery disease and rheumatoid arthritis: A systematic review and meta-analysis

**DOI:** 10.1097/MD.0000000000019658

**Published:** 2020-04-03

**Authors:** Hong Wang, Xinxin Li, Guoping Gong

**Affiliations:** Department of Cardiology, The People's Hospital of Guangxi Zhuang Autonomous Region, Nanning, Guangxi, P. R. China.

**Keywords:** cardiovascular outcomes, coronary artery disease, mortality, percutaneous coronary intervention, revascularization, rheumatoid arthritis

## Abstract

**Background:**

Through this analysis, we aimed to systematically compare the cardiovascular outcomes observed in patients with co-existing coronary artery disease (CAD) and rheumatoid arthritis (RA).

**Methods:**

Mendeley, Web of Science (WOS), MEDLINE, Cochrane central, EMBASE, Google scholar, and http://www.ClinicalTrials.gov were searched for English-based publications on CAD and RA. Selective cardiovascular outcomes were the endpoints in this analysis. The statistical software RevMan 5.3 was used for data assessment. Risk ratios (RR) with 95% confidence intervals (CI) were used to represent each subgroup analysis.

**Results:**

One thousand four hundred forty six (1446) participants had co-existing CAD and RA whereas 205,575 participants were in the control group (only CAD without RA). This current analysis showed that the risk of asymptomatic or stable angina was similar in CAD patients with versus without RA (RR: 0.98, 95% CI: 0.84 – 1.14; *P* = .78). However, all-cause mortality (RR: 1.47, 95% CI: 1.34 – 1.61; P = 0.00001), cardiac death (RR: 1.51, 95% CI: 1.05 – 2.17; *P* = .03) and congestive heart failure (RR: 1.41, 95% CI: 1.27 – 1.56; *P* = .00001) were significantly higher in CAD patients with RA. However, multi-vessel disease (RR: 2.03, 95% CI: 0.57 – 7.26; *P* = .28), positive stress test (RR: 1.69, 95% CI: 0.70 – 4.08; *P* = .24), and ischemic events (RR: 1.18, 95% CI: 0.81 – 1.71; *P* = .40) were similar in both groups. The risk for myocardial infarction, repeated revascularization, and the probability of patients undergoing percutaneous coronary intervention (PCI) (RR: 1.20, 95% CI: 0.75 – 1.93; *P* = .45) were also similar in CAD patients with versus without RA. When we considered outcomes only in those patients who underwent revascularization by PCI, all-cause mortality (RR: 1.43, 95% CI: 1.29 – 1.60; *P* = .00001) was still significantly higher in CAD patients with RA.

**Conclusions:**

This analysis showed a significantly higher mortality risk in CAD patients with RA when compared to the control group. Congestive heart failure also significantly manifested more in CAD patients with co-existing RA. However, the risks all the other cardiovascular outcomes were similar in both groups. Nevertheless, due to the several limitations of this analysis, this hypothesis should be confirmed in forthcoming trials based on larger numbers of CAD patients with co-existing RA.

## 1 Introduction

Rheumatoid arthritis (RA) is a progressive disabling autoimmune disease that causes inflammation, swelling, and pain around the joints and other organs of the body.^[1]^ Recent scientific investigations have shown a link between coronary artery disease (CAD) and RA.^[2]^ RA has shown to increase the risk of CAD.^[3]^ This might partly be explained by an accelerated atherogenic process involved in the pathogenesis related to immune response in the mechanism of RA and the implication of inflammation has now well been established in the development, progression and complication of CAD in RA.^[4]^ An evidence of subclinical CAD also showed recent onset RA to cause an increase in the wall thickness of left anterior descending artery and this wall thickness was shown to be linked with the disease activity.^[5]^ Since data concerning the impact of RA on CAD are still scarce and since cardiovascular outcomes have rarely been assessed in patients with RA through meta-analyses, we aimed to systematically compare the cardiovascular outcomes observed in patients with co-existing CAD and RA.

## 2 Methods

### 2.1 Searched databases

Common online searched databases including Mendeley, Web of Science (WOS), MEDLINE: a service of the National Library of Medicine, Cochrane central, EMBASE, Google scholar, and http://www.ClinicalTrials.gov were searched for English-based publications on CAD and RA. The PRISMA reporting guideline^[6]^ was followed for this systematic review and meta-analysis.

### 2.2 Searched strategies

A thorough search was carried out through the above-mentioned databases for English-based publications comparing the cardiovascular outcomes observed in CAD patients with versus without co-existing RA using the following terms:

(a) RA and CAD;
(b) RA and cardiovascular disease;
(c) RA and percutaneous coronary intervention (PCI);
(d) RA and atherosclerosis;
(e) RA and angioplasty;
(f) RA and heart disease;
(g) RA and acute coronary syndrome;
(h) RA and myocardial infarction;
(i) Rheumatism and CAD;
(j) Rheumatism and cardiovascular disease;
(k) Rheumatism and PCI;
(l) Autoimmune disorders and CAD;
(m) Autoimmune disorders and PCI.

During this search process, short abbreviations such as ACS denoting acute coronary syndrome, CAD, RA, PCI, denoting PCI, CVD denoting cardiovascular disease were also substituted with their respective full terms.

### 2.3 Inclusion and exclusion criteria

The criteria for inclusion were listed below:

(1) Studies which were randomized trials or observational studies dealing with the comparison of cardiovascular outcomes in CAD patients with versus without co-existing RA;
(2) Similar criteria as for (1) but also involving revascularization by PCI;
(3) Involved an experimental group (CAD participants with co-existing RA) and a control group (CAD participants without co-existing RA);
(4) Implicated with data which were relevant and which could be used in this current analysis.

The criteria for exclusion were:

(1) Systematic reviews of literatures/case studies/letters of correspondence/other reviews;
(2) Non-English publications;
(3) The correct cardiovascular outcomes were not reported;
(4) A control group was absent for comparison;
(5) Irrelevant data were involved and they could not be used for this current meta-analysis;
(6) Duplicated studies.

### 2.4 Types of participants, outcomes and follow-up time periods

In this study, CAD patients with and without co-existing RA were included. Also, a subgroup of participants who underwent PCI was also included.

In Table 1, the outcomes which were reported in the original studies were listed. Based on this list, we selected a few endpoints which could be assessed in this analysis:

(a) Asymptomatic or stable angina;
(b) All-cause mortality;
(c) Cardiac death;
(d) Congestive heart failure;
(e) Occurrence of multi-vessel disease;
(f) Positive stress test;
(g) Ischemic events;
(h) Myocardial infarction;
(i) Repeated revascularization;
(j) Probability of patients undergoing PCI.

**Table 1:**
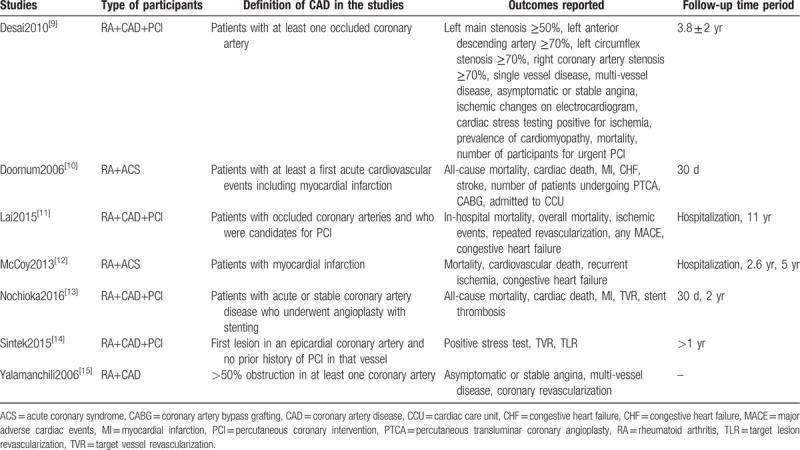
Outcomes which were reported in the original studies.

The average follow-up time periods in each studies have been given in Table 1.

### 2.5 Data extraction and quality assessment

Following the confirmation of the selected publications, data were carefully extracted by 3 independent authors. First of all, the authors confirmed whether the studies reported cardiovascular outcomes in patients with co-existing CAD and RA. Next, the total number of participants with and without RA were extracted and recorded. The corresponding outcomes and follow-up time periods were also extracted from the original studies. Then, general features including the enrollment period of the participants, the type of study involved, as well as the methodological features reported in each original study were extracted. Later on, the baseline characteristics including the mean age, the total percentage of female participants, the mean percentage of participants with diabetes mellitus, hypertension and dyslipidemia were extracted and recorded. At last, the total number of events, that is, the total number of participants involved in each particular subgroup were recorded.

The assessment of the methods in the original studies was carried out by the Cochrane collaboration^[7]^ for the randomized trials and the Newcastle Ottawa Scale (NOS)^[8]^ for the observational studies and grades (A, B or C) were allotted based on the risk of bias reported (low, intermediate or high bias).

### 2.6 Statistical analysis

The statistical software RevMan 5.3 was used to carry out this meta-analysis. Risk ratios (RR) with 95% confidence intervals (CI) were used to represent the analysis associated with each subgroup. A subgroup analysis associated with a *P* value less or equal to .05 was considered as statistically significant whereas a *P* value greater than .05 implied that there was no statistical difference between the experimental and the control group.

Heterogeneity was assessed by the *I*^2^ statistic test. The *I*^2^ value was reported in percentage (%). Heterogeneity increased with an increasing *I*^2^ value. The choice between the use of a fixed or a random statistical model was also dependent on the *I*^2^ value. In this analysis, if an *I*^2^ value was above 50%, a random effects model was used, or else, a fixed effect model was used during analysis.

Sensitivity analysis was also done to show any influence of 1 particular study on the results of this analysis. Publication bias was also visually estimated and assessed through funnel plots.

### 2.7 Ethical approval

This is a meta-analysis involving data which were extracted from previously published original studies. Therefore, no ethical approval was required since the authors were not involved in carrying out experiments on animals or humans for this analysis.

## 3 Results

### 3.1 Searched outcomes

Our searched outcomes resulted in a total number of 657 publications. The 3 authors carefully assessed the titles of these publications and carefully read the abstracts to look for the content and data. Based on this initial assessment, 586 publications were eliminated since they were irrelevant and fell outside the aim and scope of this research paper. Seventy-one full text publications were assessed for eligibility based on the inclusion and exclusion criteria.

Another assessment was carried out on the 71 full text articles and then studies were eliminated due to the following reasons:

(1) They were literature reviews/systematic reviews/case studies/letters of correspondence/other reviews (n = 19);
(2) They did not involve a control group (n = 7);
(3) They were non-English publications (n = 4);
(4) They did not report the relevant cardiovascular outcomes (n = 3);
(5) Their data could not be used in this analysis (n = 3);
(6) They were duplicated/repeated studies (n = 28).

**Figure 1:**
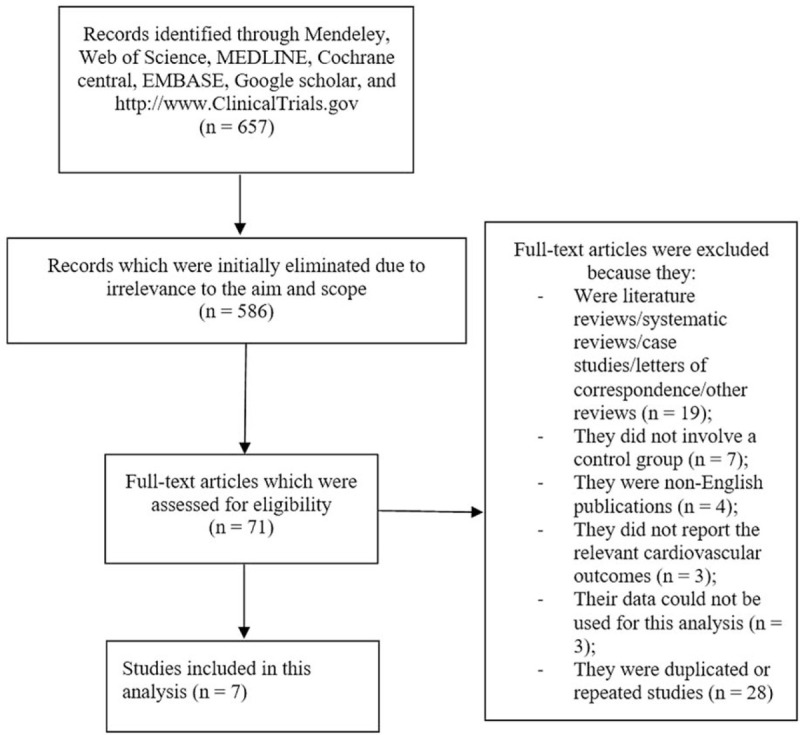
Flow diagram representing the selection of studies for inclusion in this analysis.

At last, we remained with 7 studies (9 – 15) which were finally selected for this meta-analysis (Fig. 1).

### 3.2 General and baseline features of the studies

One thousand four hundred forty six participants had co-existing CAD and RA whereas 205,575 participants were in the control group (CAD without RA) as shown in Table 1. In total, there were 207,021 participants enrolled between year 1979 and 2012 in this analysis.

The methodological quality of these original studies were assessed. A grade B signifying moderate risk of bias was allotted to each of these studies.

**Table 2:**
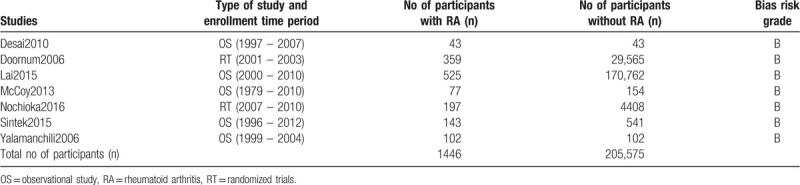
Main features of the studies involved.

The baseline features (Table 1) were: A mean age of the participants lied between 63.1 and 74.8 years. The mean percentage of female patients varied between 41% and 66.9% in the RA group and 23% to 64% in the non-RA group as shown in Table 3. The percentage of patients with co-existing diabetes mellitus ranged between 13.1% and 40.0%, whereas the percentage of patients with hypertension and dyslipidemia ranged between 30.4% and 91.0% and 4.70% and 81.0% respectively (Table 3).

**Table 3:**
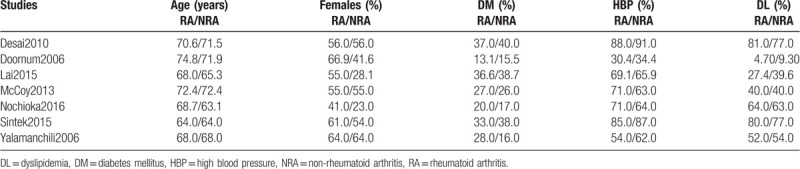
Baseline features of the studies involved.

The medication history (including drugs prescribed at discharge) has been listed in Table 4. It should be noted that medication history listed in the original papers were mentioned. If medications were not listed in the original studies, they were not mentioned in the Table.

**Table 4:**
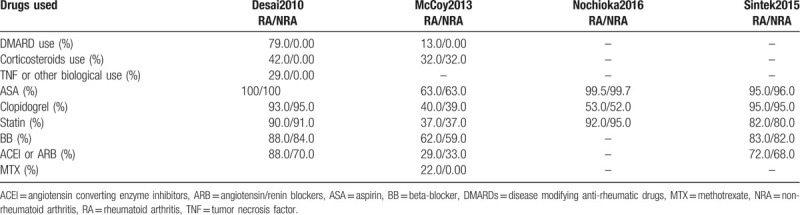
Medication use by the participants.

### 3.3 Cardiovascular outcomes in patients with co-existing CAD and RA

**Figure 2:**
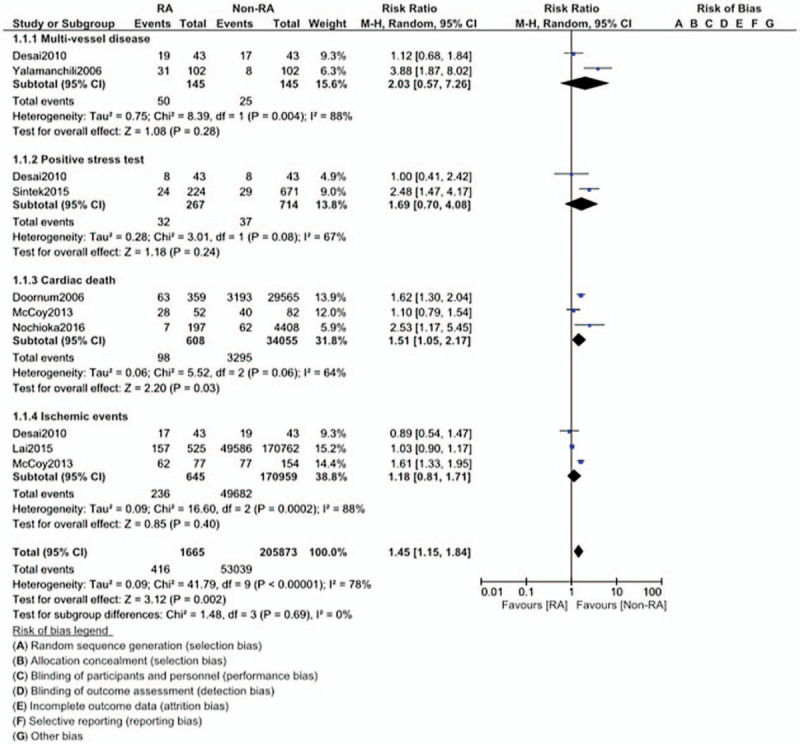
Comparison of cardiovascular outcomes in CAD patients with versus without rheumatic arthritis (Part I).

This current analysis showed that the risk of asymptomatic or stable angina was similar in CAD patients with versus without RA (RR: 0.98, 95% CI: 0.84 – 1.14; *P* = .78). However, all-cause mortality (RR: 1.47, 95% CI: 1.34 – 1.61; *P* = .00001) and congestive heart failure (RR: 1.41, 95% CI: 1.27 – 1.56; *P* = .00001) were significantly higher in CAD patients with RA as shown in Figure 2.

**Figure 3:**
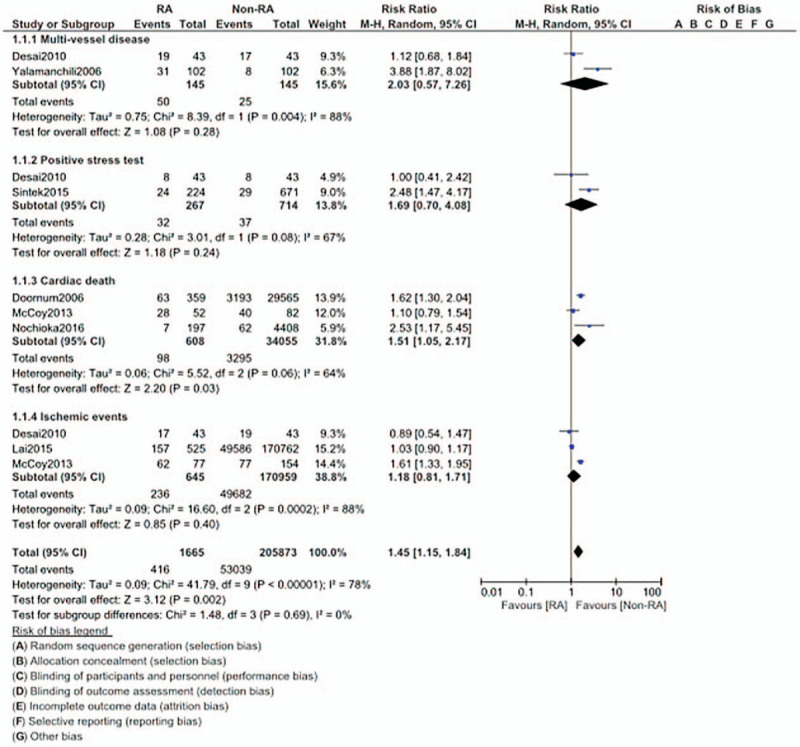
Comparison of cardiovascular outcomes in CAD patients with versus without rheumatic arthritis (Part II).

Cardiac death (RR: 1.51, 95% CI: 1.05 – 2.17; *P* = .03) was also significantly higher in CAD patients with RA. However, our analysis showed that multi-vessel disease (RR: 2.03, 95% CI: 0.57 – 7.26; *P* = .28), positive stress test (RR: 1.69, 95% CI: 0.70 – 4.08; *P* = .24), and ischemic events (RR: 1.18, 95% CI: 0.81 – 1.71; *P* = .40) were similar in both groups as shown in Figure 3.

**Figure 4:**
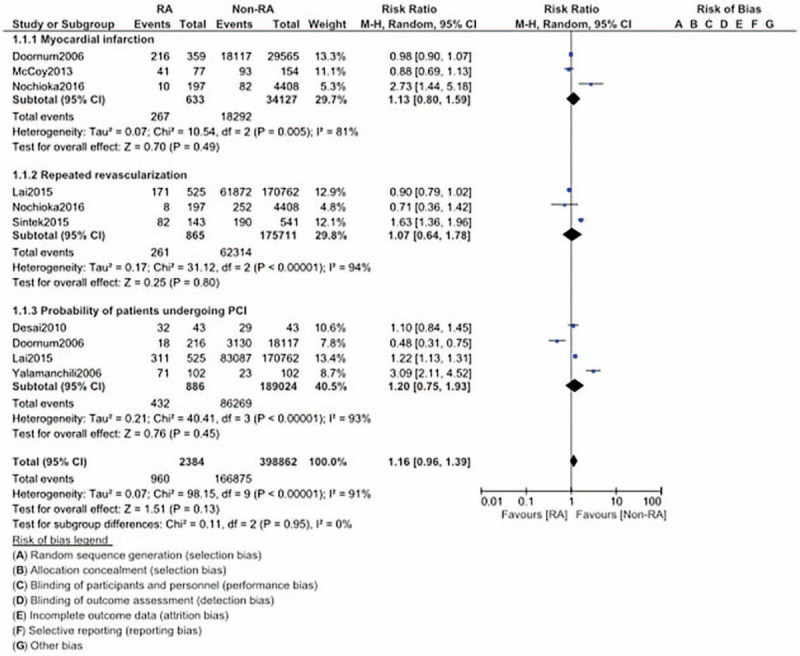
Comparison of cardiovascular outcomes in CAD patients with versus without rheumatic arthritis (Part III).

The risk for MI (RR: 1.13, 95% CI: 0.80 – 1.59; *P* = .49), repeated revascularization (RR: 1.07, 95% CI: 0.64 – 1.78; *P* = .80), and the probability of patients undergoing PCI (RR: 1.20, 95% CI: 0.75 – 1.93; *P* = .45) were also similar in CAD patients with versus without RA as shown in Figure 4.

**Figure 5:**
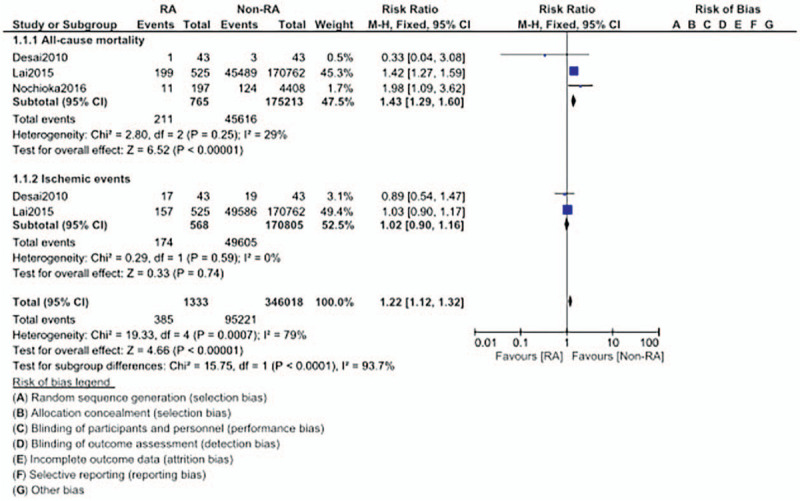
Comparison of cardiovascular outcomes in CAD patients with versus without rheumatic arthritis (Part IV).

When we considered outcomes only in those patients who underwent revascularization by PCI, all-cause mortality (RR: 1.43, 95% CI: 1.29 – 1.60; *P* = .00001) was still significantly higher in CAD patients with co-existing RA as shown in Figure 5. However, ischemic events (RR: 1.02, 95% CI: 0.90 – 1.16; *P* = .74) manifested at a similar rate.

**Figure 6:**
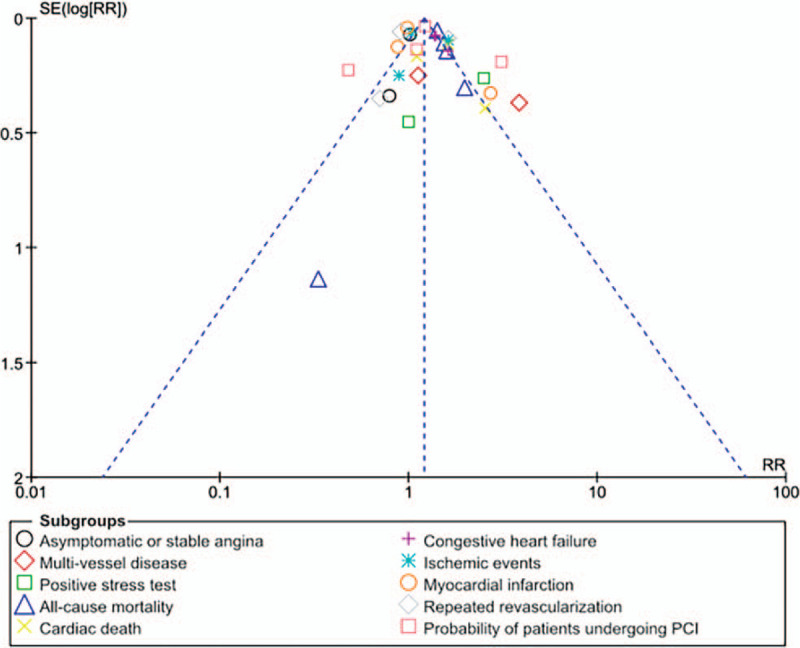
Funnel plot showing the visual assessment of publication bias.

Based on a sensitivity analysis, a consistent result was obtained throughout and publication bias was demonstrated through the funnel plot represented by Figure 6.

A summarized version of the overall results of this current analysis has been given in Table 5.

**Table 5:**
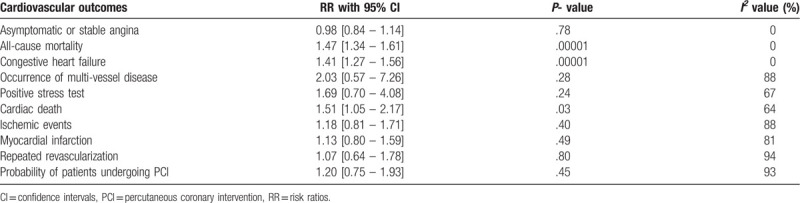
Results of this analysis comparing the cardiovascular outcomes in patients with co-existing coronary artery disease and rheumatoid arthritis.

## 4 Discussion

This current analysis showed mortality, including all-cause death and cardiac death, as well as congestive heart failure to be significantly higher in CAD patients with co-existing RA when compared to the control group. However, other outcomes including repeated revascularization and ischemic events and even patients undergoing revascularization by PCI were similar in both groups.

Results from a large population based cohort in Sweden showed mortality to have been decreased in patients with RA but, unfortunately, an excess risk of death was still observed in CAD patients with co-existing RA,^[16]^ further supporting this current analysis. In addition, in a meta-analysis including 24 observational studies with a total number of 111,758 participants, 22,927 cardiovascular mortality occurred showing a 50% increased risk of death in patients with RA.^[17]^

Based on the data reported in each study included in this current analysis, and an overall analysis of mortality, our current results showed a higher rate of all-cause mortality and cardiac death among patients with RA. To provide an explanation to such an outcome, recent studies have shown this high rate of mortality to be due to endothelial dysfunction^[18]^ and due to circulating acute phase reactants such as C reactive proteins,^[19]^ which are apparently elevated in patients with RA further worsening chronic inflammations.

Further explanations are related to the involvement of biological mediators in patients with RA which have shown to exacerbate inflammatory reactions and further worsen CAD.^[20]^ A study based on the associations between IL-6 and echo-parameters in patients with early onset CAD showed plasma IL-6 level to be associated with other inflammatory parameters and the function of the heart, resulting in left ventricular systolic dysfunction and right ventricular remodeling.^[21]^ Other chronic inflammatory mediators such as microRNAs including miR-21 and miR-146 were significantly increased in patients with vulnerable coronary plaques showing a close association between those chronic inflammatory mediators and CAD.^[22]^ MicroRNA has shown to also play an important role in the pathophysiology of atherosclerosis, by mediating intercellular crosstalk, modulating vascular smooth muscle and endothelial cells following injury to vessels.^[23]^

Nevertheless, in a structured medical chart review of RA patients,^[24]^ it was seen that RA patients received less treatment with beta blockers and lipid lowering agents in the hospital and this might be the reasons contributing to the higher risk of mortality in RA patients. An Italy-based study showed that among RA patients with concomitant CAD, revascularization by PCI was associated with significantly higher risks of long-term major adverse cardiac events.^[25]^ In addition, drugs such as corticosteroids which have been used in the treatment of RA, might elevate cardiovascular risk factors and exacerbate heart diseases by raising the blood lipid levels as well as the blood pressure.^[26]^

Our current analysis did not show any increase in the risk of multi-vessel disease due to a limited number of studies reporting this feature. However, a retrospective case-control study of Olmsted County United States of America,^[27]^ showed that RA patients with recent onset CAD, an increased risk of multi-vessel CAD was observed in that specific population of patients. The study also showed the implication of T cells to the progression of atherosclerosis in these patients.

In another 41 RA patients, postmortem autopsy revealed that grade of occlusion and the number of acute coronary lesions were similar to those who did not have RA,^[28]^ but among those who had RA and cardiovascular disease, they were more prone to vulnerable plaques in the left anterior descending artery.

In this current analysis, patients who required revascularization by PCI were similar to those who had co-existing RA versus patients who did not have RA. However, in a cross sectional analysis including a total number of 1112676 patients with myocardial infarction, a higher number of patients with RA was seen in the thrombolytic and PCI groups.^[29]^

Nevertheless, in a pilot study involving Asian patients with RA, the authors concluded that CAD was not increased in patients with RA, or simply RA was not linked with an increase in CAD.^[30]^ Also, among participants from the National Health Insurance Research Database who underwent non-stenting PCI,^[31]^ no significant difference was observed in in-hospital mortality and re-admission at 1 year in patients with RA. A study even mentioned an in-hospital survival advantage among RA participants and reduced hospital stay following revascularization.^[32]^ In another cross-sectional study, the incidence and progression of coronary artery calcium did not differ in patients with versus without RA.^[33]^

Even though mortality was higher in CAD patients with co-existing RA, the fact that medications such as statin, methotrexate, aspirin and tumor necrosis factor inhibitors might instead prevent cardiovascular damages should not be ignored. Statin has shown to reduce vascular inflammation, and atorvastatin has shown to mildly decrease RA disease activity, improve arterial stiffness and endothelial dysfunction therefore protecting the heart.^[34]^ Aspirin has also shown to be beneficial and has often been prescribed as prophylaxis for patients with CAD.^[35]^ Methotrexate, which is a first line medication for the treatment of RA, has shown to significantly reduce cardiovascular risk.^[36]^ Tumor necrosis factor inhibitors on the other hand, elevate high density lipoproteins in blood therefore, they might stabilize atherogenic ratio indirectly protecting the heart.^[37]^

## 5 Limitations

The limitations were: This analysis included only 1446 participants with co-existing CAD and RA. This total number of participants in the experimental group was less when compared to the total number of participants in the control group. Secondly, in this analysis, the follow-up time period was ignored and that might affect the results to a minimal extent. Another limitation might be the fact that several cardiovascular subgroups involved the inclusion of only 2 studies for analysis since the outcomes associated with those subgroups were not reported in the other studies. Moreover, heterogeneity level was quite high during analysis of several subgroups due to the inclusion of data obtained from observational studies. This might also have affected the results. Another limitation might be the fact that the anti-rheumatoid medications, as well as the duration of disease were ignored in this analysis. Also, even if our current analysis showed an increase in the rate of congestive heart failure among RA patients, there was no data relevant to medication compliance or under-utilization of cardiac medications such as beta-blockers, angiotensin converting enzyme inhibitors, aspirin, clopidogrel, or statin in these patients representing another limitation of this analysis. Data concerning the use of methotrexate were given in only 1 study, and this was not sufficient to show any positive impact of this medication on outcomes. Moreover, due to limited data given in the original studies, it was also not clear whether those patients with RA were in their active or controlled disease state.

## 6 Conclusions

This analysis showed a significantly higher mortality risk in CAD patients with RA when compared to the control group. Congestive heart failure also significantly manifested more in CAD patients with co-existing RA. However, the risks all the other cardiovascular outcomes were similar in both groups. Nevertheless, due to the several limitations of this analysis, this hypothesis should be confirmed in forthcoming trials based on larger numbers of CAD patients with co-existing RA.

## Acknowledgment

There was no any other person who was involved in any work related to the preparation or revision of this manuscript.

## Author contributions

Dr Hong Wang, Xinxin Li and Guoping Gong were responsible for the conception and design, acquisition of data, analysis and interpretation of data, drafting the initial manuscript and revising it critically for important intellectual content. Dr Hong Wang wrote this manuscript.

**Conceptualization:** Hong Wang, Xinxin Li, Guoping Gong.

**Data curation:** Hong Wang, Xinxin Li, Guoping Gong.

**Formal analysis:** Hong Wang, Xinxin Li, Guoping Gong.

**Funding acquisition:** Hong Wang, Xinxin Li, Guoping Gong.

**Investigation:** Hong Wang, Xinxin Li, Guoping Gong.

**Methodology:** Hong Wang, Xinxin Li, Guoping Gong.

**Project administration:** Hong Wang, Xinxin Li, Guoping Gong.

**Resources:** Hong Wang, Xinxin Li, Guoping Gong.

**Software:** Hong Wang, Xinxin Li, Guoping Gong.

**Supervision:** Hong Wang, Xinxin Li, Guoping Gong.

**Validation:** Hong Wang, Xinxin Li, Guoping Gong.

**Visualization:** Hong Wang, Xinxin Li, Guoping Gong.

**Writing – original draft:** Hong Wang.

**Writing – review & editing:** Hong Wang.
